# Temporal Expression Pattern of Hemoxygenase-1 Expression and Its Association with Vasospasm and Delayed Cerebral Ischemia After Aneurysmal Subarachnoid Hemorrhage

**DOI:** 10.1007/s12028-021-01299-w

**Published:** 2021-07-26

**Authors:** Sibylle Frase, Matti Steimer, Lisa Selzner, Sandra Kaiser, Niels Alexander Foit, Wolf-Dirk Niesen, Nils Schallner

**Affiliations:** 1 Department of Neurology and Neuroscience, Medical Center - University of Freiburg, Freiburg, Germany; 2 Faculty of Medicine, University of Freiburg, Freiburg, Germany; 3 Department of Anesthesiology and Critical Care, Medical Center - University of Freiburg, Freiburg, Germany; 4 Department of Neurosurgery, Medical Center - University of Freiburg, Freiburg, Germany

**Keywords:** Subarachnoid hemorrhage (aneurysmal), Vasospasm (intracranial), Biomarkers, Heme oxygenase, Outcome assessment

## Abstract

**Background:**

Red blood cell-induced cerebral inflammation and toxicity has been shown to be attenuated by induction of the heme-catalyzing enzyme, hemoxygenase-1 (HO-1), in animal models of subarachnoid hemorrhage (SAH). Although inflammatory mechanisms leading to secondary neuronal injury in SAH are becoming increasingly well understood, markers of cerebral inflammation have so far not been implemented in clinical prediction models of SAH.

**Methods:**

In this biomarker observational study, *HO-1* messenger ribonucleic acid (mRNA) expression levels were determined in cerebrospinal fluid (CSF) and blood of 66 patients with aneurysmal SAH on days 1, 7, and 14 after the SAH event. *HO-1* mRNA expression was determined via real time polymerase chain reaction (PCR), and relative expression changes were quantified in comparison with expression levels in nonhemorrhagic control CSF. Subarachnoid blood burden, as well as presence of vasospasm and delayed cerebral ischemia (DCI), were recorded. Short and long-term clinical outcomes were assessed using the Modified Rankin Scale at discharge and 1 year after the SAH event.

**Results:**

CSF *HO-1* expression levels showed a significant increase over the 14-day observation period (*p* < 0.001, *F* = 22.53) and correlated with intracranial hematoma burden (*ρ* = 0.349, *p* = 0.025). In multivariate analyses, CSF *HO-1* expression levels did not reach significance as independent predictors of outcome. Vasospasm on computed tomographic angiography was associated with lower CSF *HO-1* expression levels on day 7 after SAH (*n* = 53, *p* = 0.010), whereas patients with DCI showed higher CSF *HO-1* expression levels on day 14 after SAH (*n* = 21, *p* = 0.009).

**Conclusions:**

*HO-1* expression in CSF in patients with SAH follows a distinct temporal induction pattern and is dependent on intracranial hematoma burden. CSF *HO-1* expression was unable to predict functional outcome. Associations of early low *HO-1* expression with vasospasm and late elevated *HO-1* expression with DCI may point to detrimental effects of late *HO-1* induction, warranting the need for further investigation in a larger study population.

## Introduction

Aneurysmal subarachnoid hemorrhage (SAH) continues to be associated with high mortality and persisting disability rates [1], even more so in poor-grade SAH (Hunt & Hess or World Federation of Neurosurgical Societies [WFNS] grades 4 and 5) [2]. Even patients with favorable outcome [Modified Rankin Scale (mRS) < 3] frequently experience long-term cognitive impairment and decreased quality of life despite functional independence [3]. In addition to early brain injury induced by the acute hemorrhage, secondary complications, including vasospasm, hydrocephalus, and delayed cerebral ischemia (DCI), contribute substantially to long-term disability.

Over the past decades, it has become increasingly evident that, although the presence of vasospasm is associated with DCI and increased mortality and morbidity [4, 5], its successful treatment does not improve clinical outcome [6]. It has been postulated that angiographic vasospasm is merely one of multiple sequelae of red blood cell (RBC)-induced cerebral inflammation, whereas microthrombosis, impaired cerebral autoregulation, peripheral vasospasm, and cortical spreading depression represent other contributors to DCI [7–9], and that treatment of vasospasm alone is insufficient because it does not address the underlying inflammatory pathology [10]. Furthermore, oxidative stress has been associated with poor outcome and greater incidence of vasospasm after SAH [11, 12], whereas the attenuation of oxidative injury reduced neuronal apoptosis following SAH in a preclinical model [13].

Following aneurysm rupture, free RBC components in the subarachnoid space induce neuroinflammation, perturbations of the blood–brain barrier, and mediate direct cytotoxic effects on central nervous system cells, leading to edema [14–16]. The degradation of free heme into biliverdin, iron, and carbon monoxide (CO) is catalyzed by hemoxygenase enzymes (HO). The isoform hemoxygenase-1 (HO-1) is rapidly induced in models of traumatic brain injury, intracerebral and subarachnoid hemorrhage [17, 18]. Induction of microglial HO-1 has not only been shown to mediate the clearance of subarachnoid blood but also to attenuate neuronal injury and vasospasm in animal models after SAH [19–21]. HO-1 has also been demonstrated to exert protective effects against neuronal ischemia independently of intracranial hemorrhage (ICH) [22]. In patients with SAH, cerebrospinal fluid (CSF) *HO-1* mRNA (messenger ribonucleic acid) expression levels were significantly elevated [19, 20] relative to controls without ICH. *HO-1* expression levels also correlated with intracranial hematoma volume, underlining the importance of *HO-1* activity for the clearance of subarachnoid blood [19]. In humans, the temporal pattern of CSF *HO-1* expression after SAH has not been studied before. Because *HO-1* is predominantly expressed in microglia after ICH [23, 24], the characterization of expression patterns could provide insight into neuroinflammatory processes.

Other markers of neuroinflammation have also been shown to be elevated even systemically. For instance, serum levels of the proinflammatory damage-associated molecular pattern molecule high-mobility group box-1 were elevated only in patients with SAH who developed vasospasm [25]. CSF interleukin-6 levels, although elevated in all patients with SAH, were significantly higher in patients who developed vasospasm [26, 27].

Despite growing understanding of the contribution of inflammation to secondary neuronal injury in SAH, markers of neuroinflammation have so far not been implemented in prediction and risk assessment in the clinical setting. Grading of SAH severity is predominantly based on neurological symptoms at disease onset and on the extent of subarachnoid blood burden on imaging [28–30].

The objective of this single-center observational study was to examine whether CSF and blood *HO-1* mRNA expression levels of patients with SAH are indicators for intracranial hematoma burden and disease severity, and whether early measurement of *HO-1* expression levels could predict short-term and long-term clinical outcomes, as sufficient HO-1 activity was associated with better functional outcome in murine models of SAH. Moreover, we sought to examine whether early measurement of *HO-1* expression could identify patients at risk for secondary neurological deterioration after the initial bleeding event.

## Methods

### Study Design

A total of 66 patients with spontaneous SAH (19 men, 47 women, aged 58.03 ± 13.22 years, range 27–88 years, Table 1) admitted to either the intensive care units (ICUs) of the Department of Neurology and Neuroscience or the Department of Neurosurgery at the University of Freiburg (Germany) Medical Center between 2017 and 2020 were included in this study. The study protocol was approved by the Institutional Ethics Review Board of the University of Freiburg (Protocol no. 293/15). Informed consent from the patient, legal guardian, or by proxy was provided. The trial was registered with the German Clinical Trials Register (Trial-ID DRKS00008981; Universal Trial Number U1111-1172-6077).

**Table 1:** Patient characteristics

	Mean ± SD	*n*
Age (years)	58.0 ± 13.2	66
Modified Rankin Scale		
On admission	4.3 ± 1.0	66
At discharge	3.4 ± 1.7	66
1 year after SAH	2.7 ± 2.2	50
SAH severity scales		
Hunt and Hess grade	3.2 ± 1.1	66
WFNS grade	3.3 ± 1.4	64
Modified Fisher grade	3.7 ± 0.7	63
Total hematoma volume (cm^3^)	22.9 ± 22.8	52
Hijdra Sum Score	24.2 ± 9.2	62

*SAH* subarachnoid hemorrhage, *SD* standard deviation, *WFNS* World Federation of Neurosurgical Societies

The following criteria for inclusion were applied:

- Age > 18 years.
- Spontaneous SAH confirmed on computed tomography (CT) scan or via lumbar puncture/CSF xanthochromia.
- Admission to the ICU and placement of an external ventricular drain (EVD) for therapeutic purposes, as well as first CSF and blood sample collection within 24 h after the SAH event.
- Provision of informed consent from the patient, legal guardian, or by proxy.

The following criteria for exclusion were applied:

- Age < 18 years.
- Admission later than 24 h after symptom onset.
- Current pregnancy.
- Death within 24 h of ICU admission.
- Evidence of septic aneurysm origin or evidence of ventriculitis or meningitis during the time period of sample collection.
- Evidence of subdural or epidural hematoma on initial CT imaging.

### Sample Collection and Analysis

**Fig. 1:**
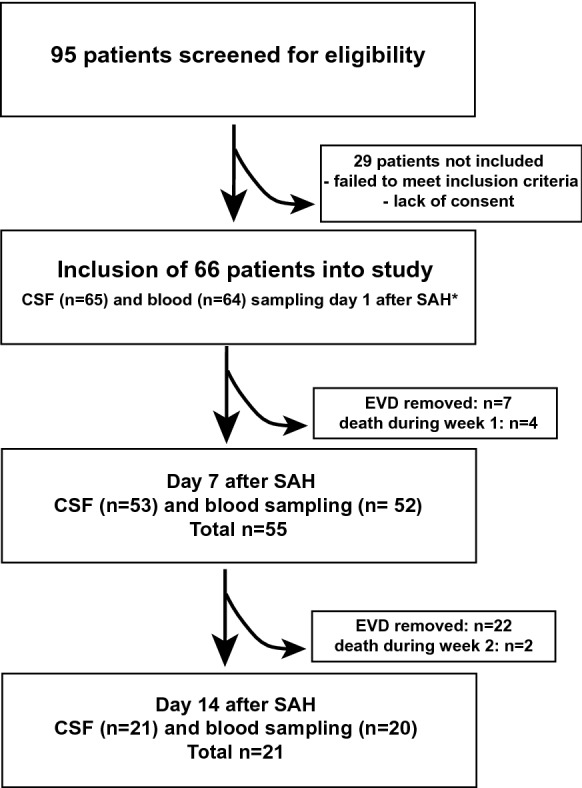
Schematic depiction of the study design. *Biomaterial was partially insufficient for analysis in three patients (one CSF and two blood samples on day 1 after SAH). CSF, cerebrospinal fluid, EVD, external ventricular drain, SAH, subarachnoid hemorrhage

Cerebrospinal fluid and blood samples were obtained from patients with SAH on days 1, 7, and 14 after SAH symptom onset. Blood samples were obtained from either arterial or central venous catheters, whereas CSF samples were acquired under sterile conditions from EVDs placed for therapeutic purposes. If the patients’ external ventricular drain and/or arterial or venous catheters were removed because of clinical improvement or other reasons before day 7 or 14 after SAH symptom onset, sample collection was terminated (Fig. 1).

*HO-1* mRNA expression levels from CSF and peripheral blood samples were determined as previously described.

Blood samples were stored at − 80 °C in RNA stabilizing reagent tubes (Tempus Blood RNA Tube, AB#4342792). RNA isolation from leukocytes was performed using the correspondent spin-column RNA isolation kit (Tempus Spin RNA Isolation Kit, AB#1710145). RNA content and purity were assessed photometrically (NanoDrop 2000 Spectrophotometer; Thermo Fisher Scientific Inc). RNA from CSF cells was isolated with TRIzol^TM^ and concentrated by spin-column purification (RNeasy Micro Kit; Qiagen, Hilden, Germany). Reverse transcriptase polymerase chain reaction (PCR) technique (iScript cDNA (complementary deoxyribonucleic acid) Synthesis Kit, BioRad#1708890; PeqStar 96 Universal Gradient, PeqLab) was used to reversely transcribe RNA into cDNA. Semiquantification of cDNA was performed via real time PCR (sqPCR; StepOnePlus Real Time PCR-System, A&V Applied Biosystems) with nucleic acid stain (PowerUp SYBR Green Master Mix, AB#1708020) and specific primers for *HO-1* and ribosomal protein L13a (*Rpl13a*), which served as intraindividual reference gene. CSF and blood samples of patients without ICH (patients receiving shunt surgery for normal pressure hydrocephalus) served as the interindividual reference population to calculate relative changes in mRNA expression levels. Expression levels of *HO-1* mRNA were quantified using the 2^−∆∆*Ct*^ method, in which ∆*Ct* (delta cycle threshold) = *Ct* (target gene *HO-1*) − Ct (reference gene *Rpl13a*) and ∆∆CT = ∆CT (study population) − ∆CT (reference population) [31].

Primer sequences were:

**Table:** 

*HO-1 forward*	*GTGATAGAAGAGGCCAAGACTG*
*HO-1 reverse*	*GAATCTTGCACTTTGTTGCTGG*
*Rpl13a forward*	*CGGACCGTGCGAGGTAT*
*Rpl13a reverse*	*CACCATCCGCTTTTTCTTGTC*

### Clinical Data Collection

As part of enrolment in the study protocol, and considering patients’ electronic charts and initial neuroimaging, the following clinical parameters were collected: demographic data (age and sex), SAH severity scores (Hunt & Hess grade, modified Fisher grade, WFNS grade), occurrence of sonographic or CT angiographic vasospasm during the course of ICU treatment, occurrence of DCI, and mRS score at admission, discharge, and 1 year after discharge.

Sonographic vasospasm [assessed with transcranial color-coded duplex sonography (TCCD)] was defined as mean blood flow velocity > 3 kHz (moderate)/ > 4 kHz (severe) or > 1 kHz increase compared to the examination on the previous day. TCCD results were corrected for age, vertebrobasilar system, heart rate, and hematocrit at the time of examination. Transcranial doppler sonography has been shown to have a high sensitivity (90%) to predict DCI [32]. DCI was defined as delayed onset of neurologic deterioration lasting > 24 h not explained by other causes, such as electrolyte disturbance or epileptic seizures or postictal deficits, or the presence of ischemic lesions on follow-up neuroimaging [33].

Outcomes were assessed using mRS [34] collected at hospital admission, discharge from hospital, and 1 year after SAH (assessment via telephone interview).

Radiographic total hematoma volume was assessed using OsiriX Lite software on the basis of the first CT scan after admission to the primary healthcare facility. First, cisternal hematoma volume (prepontine, interpeduncular, and ambient cisterns) was calculated from adjacent CT slices extending superiorly from the level of the caudal pons to the midbrain over a total vertical distance of 15 mm, taking slice thickness into consideration. Because cisternal hematoma volume alone often does not give an accurate representation of the total intracranial hematoma burden, ventricular and parenchymal hematoma volumes were calculated, if present, and added to the cisternal hematoma volume. In addition to this quantitative assessment, the semiquantitative Hijdra sum score [30] was determined for each patient’s initial CT scan.

Investigators performing clinical and radiographic ratings (mRS, hematoma volume) were blinded regarding PCR results and vice versa.

### Statistical Analyses

Statistical analyses were performed using GraphPad Prism software (version 8.4.3; GraphPad Software, San Diego, CA, www.graphpad.com).

According to previously published data [19], an a priori power analysis (Wilcoxon Mann–Whitney *U*-test, group comparison CSF *HO-1* expression vs. mRS at discharge favorable/nonfavorable, effect size Cohen’s w 0.8; *α* = 0.05; power 95%, *df* 2) yielded a necessary sample size of 54 patients for biomarker discriminability.

Spearman nonparametrical correlation (*ρ*, *p*) was used to assess correlation between metrical data sets (total hematoma volume, *HO-1* expression, mRS, Hunt & Hess, modified Fisher, and WFNS grades). The Mann–Whitney *U*-test was used for between-group comparisons (vasospasm yes/no, DCI yes/no, mortality yes/no, mRS 0–3 vs. 4–6, compared with *HO-1* expression) of metrical data. Receiver operating characteristic (ROC) analyses were used to explore predictive power of HO-1 expression levels regarding functional outcome, vasospasm, and DCI. A mixed-effects analysis followed by Tukey’s multiple comparisons test was used to assess tendencies in repeated measures data (mRS, *HO-1* expression). The relationship between CSF *HO-1* expression and outcome [dichotomized into mRS 0–3 (favorable) and mRS 4–6 (nonfavorable)] was explored with multiple logistic regression, with age, hematoma volume, and WFNS (grade I–III vs. IV–V) as additional independent variables. Results are displayed as median and interquartile range. In all analyses, *p* values < 0.05 were considered statistically significant.

## Results

### Total Intracranial Hematoma Volume is an Indicator of Disease Severity

**Fig. 2:**
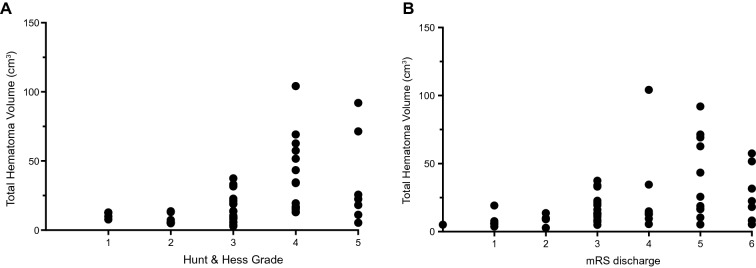
Total hematoma volume is an indicator of disease severity. **a** Total hematoma volume significantly correlated with Hunt & Hess Grade, indicating disease severity (*ρ* = 0.570, *p* < .001, *n* = 52). **b** mRS at discharge, a measure of short-term functional outcome, also correlated with total hematoma volume (*ρ* = 0.469, *p* < .001, *n* = 52). mRS, Modified Rankin Scale

Total intracranial hematoma volume showed significant correlation with Hunt & Hess Grade (Fig. 2a, *ρ* = 0.570, *p* < 0.001), modified Fisher Score (*ρ* = 0.413, *p* = 0.002), and WFNS (*ρ* = 0.625, *p* < 0.001) Grade. Total hematoma volume also significantly correlated with mRS on admission (*ρ* = 0.600, *p* < 0.0001) and discharge (Fig. 2b, *ρ* = 0.469, *p* < 0.001).

### CSF HO-1 mRNA Expression Levels Increase After SAH

**Fig. 3:**
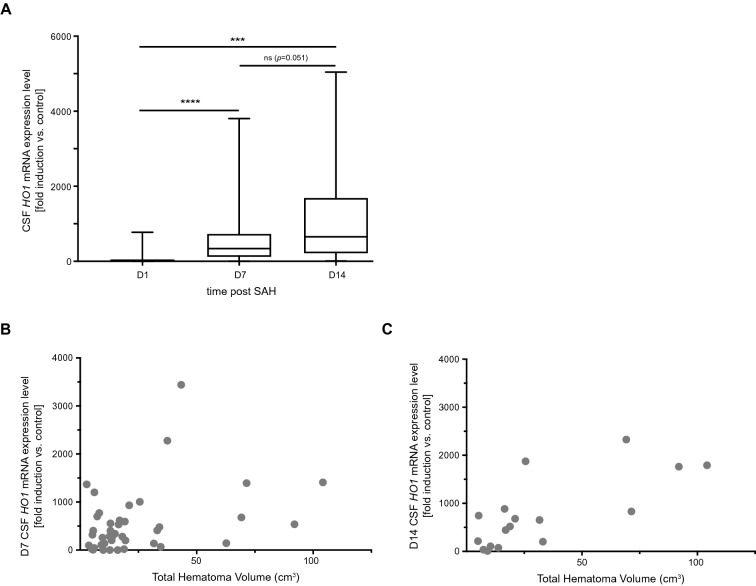
CSF *HO-1* expression is induced after the SAH event and is a function of disease severity. **a** CSF *HO-1* mRNA expression levels significantly increased after the bleeding event (*p* < 0.0001, *F* (1.692,60.92) = 22.53). **b** and **c,** *HO-1* mRNA expression levels on days 7 and 14 after SAH significantly correlated with total intracranial hematoma volume (day 7 post SAH: *ρ* = 0.349, *p* = 0.025, *n* = 41. Day 14 post SAH: *ρ* = 0.674, *p* = 0.002, *n* = 18). Column data are plotted as median with interquartile range and minimum to maximum, ***p < 0.001, ****p < 0.0001. *CSF* cerebrospinal fluid, *HO-1* hemoxygenase-1, *mRNA* messenger ribonucleic acid, *SAH* subarachnoid hemorrhage

CSF *HO-1* mRNA expression levels exhibited a significant increase during the 14-day time course of observation, indicating *HO-1* induction that markedly outlasted the initial bleeding event (Fig. 3a, *p* < 0.0001, *F*_1.692,60.92_ = 22.53; day 1 vs. day 7 post SAH: *p* < 0.0001, *n* = 53, day 1 vs. day 14 post SAH: *p* < 0.001, *n* = 21, day 7 vs. day 14 post SAH: *p* = 0.051, *n* = 21).

### CSF HO-1 mRNA Expression Correlates with Total Hematoma Volume

CSF *HO-1* mRNA expression levels on days 7 and 14, but not day 1, after SAH correlated significantly with total intracranial hematoma volume [day 1 post SAH: *ρ* = 0.056, *p* = 0.695, *n* = 51 (data not shown). Day 7 post SAH: *ρ* = 0.349, *p* = 0.025, *n* = 41, Fig. 3b. Day 14 post SAH: *ρ* = 0.674, *p* = 0.002, *n* = 18, Fig. 3c].

### Systemic HO-1 mRNA Expression is Independent of Disease Severity

**Fig. 4:**
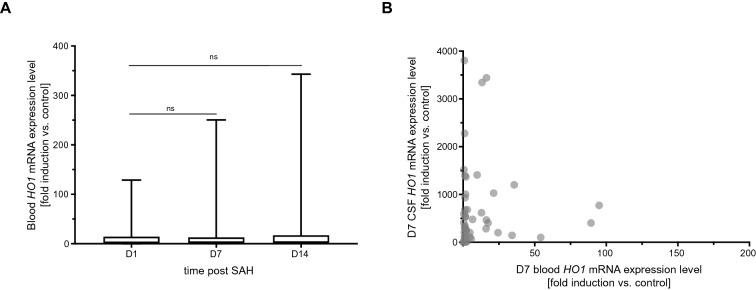
Systemic *HO-1* expression does not reflect cerebral *HO-1* induction. **a** Blood leukocyte *HO-1* mRNA expression levels did not exhibit a significant increase over the course of the disease (*p* = 0.289, *F*_1.25, 43.31_ = 1.21). **b** There was no correlation between blood and CSF *HO-1* mRNA expression levels (day 7 post SAH: *ρ* = 0.144, *p* = 0.317, *n* = 50). Column data plotted as median with interquartile range and minimum to maximum. *CSF* cerebrospinal fluid, *HO-1* hemoxygenase-1, *mRNA* messenger ribonucleic acid, *SAH* subarachnoid hemorrhage

Subarachnoid hemorrhage leads to a rapid and substantial induction of *HO-1* expression in the cerebrospinal fluid, with *HO-1* expression levels rising over a period of 2 weeks after the bleeding event, the period of observation used in this study. The question arose whether *HO-1* expression in blood leukocytes would mirror the distinct induction pattern observed in the CSF. However, *HO-1* expression in blood leukocytes was induced to a far lesser extent than in the CSF [*HO-1* expression day 7 post SAH in the CSF (fold induction vs. control): 339.3 (111.1–735.7), in blood leukocytes: 1.81 (1.16–13.14), median and interquartile range, Table 2]. There was also no significant change in blood *HO-1* expression levels over the disease course (Fig. 4a, *p* = 0.289, *F*_1.25,43.31_ = 1.21), in contrast to the marked increase observed in the CSF.

**Table 2:** *HO-1* mRNA expression levels (fold induction vs. control)

	Median	IQR	*n*
CSF *HO-1*			
Day 1	26.84	(8.60–54.56)	65
Day 7	339.3	(111.1–735.7)	53
Day 14	652.5	(207.5–1694.0)	21
Blood *HO-1*			
Day 1	1.73	(0.93–14.67)	64
Day 7	1.81	(1.16–13.14)	52
Day 14	2.10	(1.42–17.61)	20

*CSF* cerebrospinal fluid, *HO-1* hemoxygenase-1, *IQR* interquartile range, *mRNA* messenger ribonucleic acid

Also, no correlation was found between CSF and blood *HO-1* expression levels, determined at the same time points on days 7 after SAH (Fig. 4b), as well as days 1 and 14 (data not shown).

### HO-1 Expression is Not Predictive of Functional Outcome

**Fig. 5:**
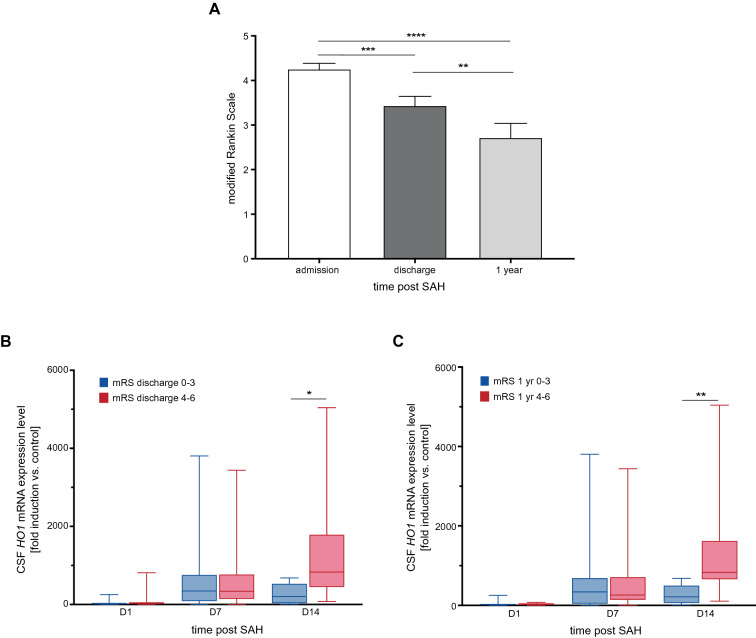
CSF *HO-1* expression levels according to short-term and long-term neurological outcome. **a** Over the course of ICU treatment and one year after the SAH event, patients showed significant clinical improvement measured by mRS (*p* < 0.0001, *F*_1.354, 77.19_ = 22.63, *n* = 50). **b** Patients with unfavorable short-term outcome showed significantly higher *HO-1* expression levels on day 14 after SAH (*n* = 21, *p* = 0.014). **c** This effect could still be observed regarding long-term functional outcome (*n* = 16, *p* = 0.008). Data are plotted as mean + SEM (mRS) and median with interquartile range and minimum to maximum. *p < 0.05, **p < 0.01, ***p < 0.001, ****p < 0.0001. *CSF* cerebrospinal fluid, *HO-1* hemoxygenase-1, *ICU* intensive care unit, *mRNA* messenger ribonucleic acid, *mRS* Modified Rankin Scale, *SAH* subarachnoid hemorrhage, *SEM* standard error of the mean

Over the course of ICU treatment and ensuing rehabilitation, patients showed significant overall clinical improvement as measured by the mRS [Fig. 5a, *p* < 0.0001, *F*_1.354, 77.19_ = 22.63; admission vs. discharge: *p* = 0.0002 (*n* = 66), admission vs. after 1 year: *p* < 0.0001 (*n* = 50), discharge vs. 1 year: *p* = 0.0013 (*n* = 50)].

The observational study design followed the natural clinical course of ICU treatment. Therefore, the endpoint of sample collection was reached once the EVD was removed because of clinical improvement (CSF samples day 1 after SAH: *n* = 65, day 7: *n* = 53, day 14: *n* = 21). This led to patients included in all 3 CSF sampling points representing a more severely affected subgroup of the total patient collective (mRS at discharge among patients with day 7 CSF samples: 3.45 ± 1.56, day 14: 4.28 ± 1.06, *p* = 0.035). 13.6% of the study population developed EVD-associated complications (ventriculitis: *n* = 5, intraparenchymal hematoma: *n* = 4).

*HO-1* mRNA expression levels on days 1 and 7 after SAH did not differ between patient groups with favorable (mRS 0–3) or unfavorable (mRS 4–6) outcome, measured at discharge from hospital (short-term, Fig. 5b) and 1 year after the SAH event (long-term, Fig. 5c). However, higher day 14 CSF *HO-1* expression levels were found in patients with unfavorable outcome at discharge from hospital and 1 year after the SAH event than in those with favorable outcome [Fig. 5b, c, mRS at discharge 0–3: 207.5 (28.05–531.7), *n* = 6, mRS at discharge 4–6: 830.9 (443.9–1792) (fold induction vs. control), *n* = 15, *p* = 0.014; mRS at 1 year 0–3: 213.4 (54.75–500.4), *n* = 9 vs. mRS at 1 year 4–6: 830.9 (652.5–1624) (fold induction vs. control), *n* = 7, *p* = 0.008]. Multiple logistic regression models for outcome (mRS at discharge favorable/nonfavorable) did not show a significant association between CSF *HO-1* expression levels on days 7 or 14 and outcome (Table 3, Model 1: mRS discharge ~ intercept + CSF *HO-1* Day 7 + WFNS grade IV–V yes/no + total hematoma volume + age, CSF *HO-1* day 7 OR 1.001 [95% confidence interval {CI} 0.99–1.00], *p* = 0.28; area under the curve (AUC) 0.83 [95% CI 0.70–0.96], *p* = 0.0004; Model 2: mRS discharge ~ intercept + WFNS grade IV–V yes/no + total hematoma volume + age, AUC 0.84 83 [95% CI 0.74–0.95], *p* < 0.0001; Model 3: mRS discharge ~ intercept + CSF *HO-1* day 14 + total hematoma volume + age, CSF *HO-1* day 14 OR 0.99 [95% CI 0.97–0.99], *p* = 0.18; AUC 0.91 [95% CI 0.77–1.0], *p* = 0.009). It should be noted that the variables CSF *HO-1* day 14 and WFNS could not both be included in one multiple logistic regression model because of multicollinearity (Spearman rank correlation *p* = 0.0008, r = 0.70). Furthermore, the number of patients still relying on an external ventricular drain 14 days after SAH was small (*n* = 21), limiting interpretability of multivariate analyses.

**Table 3:** Multiple logistic regression models with mRS favorable versus non-favorable as dependent outcome variable

	Model 1			Model 2			Model 3		
AUC	0.83	[0.70 to 0.96]	*p* = 0.0004	0.84	[0.74 to 0.95]	*p* < 0.0001	0.91	[0.77 to 1.0]	*p* = 0.009
PPP		76.47%			78.26%			80.0%	
NPP		82.61%			78.57%			92.31%	
Tjur’s *R*^2^		0.36			0.37			0.53	

Variable	OR	95% CI	*p*	OR	95% CI	*p*	OR	95% CI	*P*
Intercept	253.0	5.51 to 49,909	0.015	157.4	5.31 to 11,760	0.009	55,091	2561 to 6749e+018	0.19
CSF *HO-1* day 7	1.001	0.99 to 1.00	0.28						
CSF *HO-1* day 14							0.99	0.97 to 0.99	0.18
WFNS	0.21	0.03 to 1.17	0.09	0.14	0.03 to 0.62	0.01			
Hematoma volume	0.98	0.91 to 1.02	0.37	0.98	0.92 to 1.01	0.32	1.06	0.86 1 to 1.38	0.59
Age	0.92	0.84 to 0.98	0.03	0.94	0.88 to 0.99	0.04	0.83	0.50 to 0.99	0.18

Multiple logistic regression with mRS discharge (favorable [(0–-3]) vs. non-favorable [(4–-6])) as dependent variable. Model 1: mRS discharge ~ Intercept + CSF *HO-1* Day 7 + WFNS grade IV–/V yes/no + total hematoma volume + age. Model 2: mRS discharge ~ intercept + WFNS grade IV–/V yes/no + total hematoma volume + age. Model 3: mRS discharge ~ intercept + CSF *HO-1* day 14 + total hematoma volume + age

*AUC* area under the curve, *CI* confidence interval, *CSF* cerebrospinal fluid, *HO-1* hemoxygenase-1, *mRS* Modified Rankin Scale, *NPP* negative predictive power, *OR* odds ratio, *PPP* positive predictive power, *WFNS* World Federation of Neurosurgical Societies

### Early HO-1 Induction May Protect Against Development of Vasospasm, Whereas Late HO-1 Expression is Associated with Occurrence of DCI

**Fig. 6:**
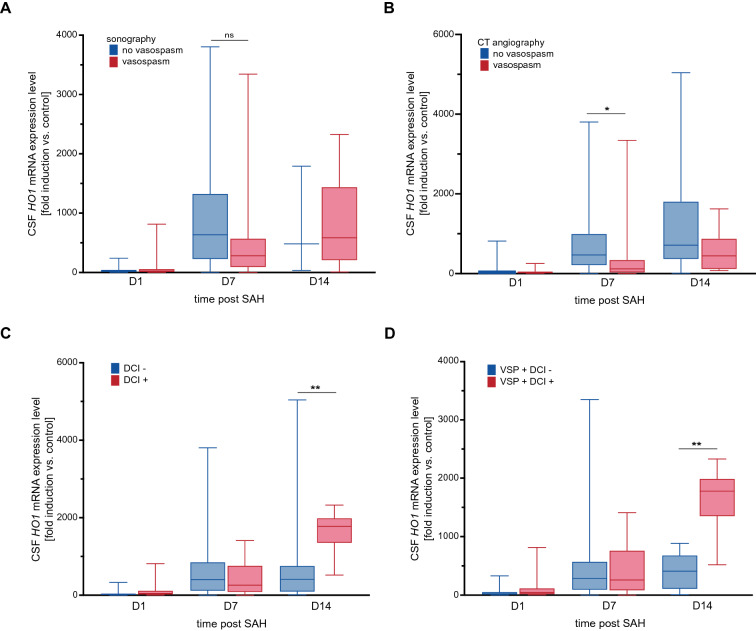
Early *HO-1* expression may influence the development of vasospasm. **a** Patients who developed sonographic vasospasm showed a trend toward lower *HO-1* mRNA expression levels on day 7 after SAH (*p* = 0.059, *n* = 50), an effect that could not be observed anymore by day 14 (*p* = 0.875, *n* = 19). **b** Irrespective of sonographic vasospasm, patients with confirmed moderate or severe vasospasm on CT angiography also showed significantly lower *HO-1* mRNA expression levels on day 7 after SAH (*p* = 0.010, *n* = 53), but not on day 14 (*p* = 0.287, *n* = 21). **c** Patients with DCI had significantly higher *HO-1* mRNA expression levels on day 14 after SAH than those without a DCI diagnosis (*p* = 0.009, *n* = 19). **d** CSF *HO-1* expression levels on day 14 were significantly higher in patients that developed vasospasm and DCI than in those that developed vasospasm but no DCI (*n* = 17, *p* = 0.002). Data are plotted as median with interquartile range and minimum to maximum. *p < 0.05, **p < 0.01. *CSF* cerebrospinal fluid, *CT* computed tomography, *DCI* delayed cerebral ischemia, *HO-1* hemoxygenase-1, *mRNA* messenger ribonucleic acid, *VSP* vasospasm, *SAH* subarachnoid hemorrhage

**Fig. 7:**
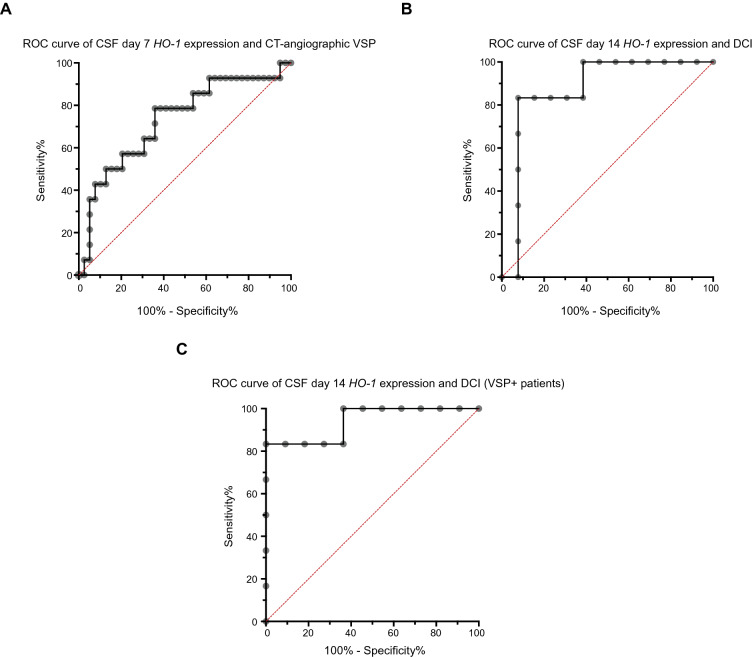
Predictive performance of CSF *HO-1* expression regarding vasospasm and DCI. **a** For the presence of vasospasm on CT angiography and *HO-1* mRNA expression on day 7, ROC curve analysis showed an AUC of 0.73 (0.57–0.89) (*p* = 0.01). **b** AUC was 0.87 (0.69–1.0) for *HO-1* expression level and presence of DCI on day 7 after SAH (*p* = 0.01). **c** In the subgroup of patients with sonographic vasospasm, ROC analysis showed an AUC of 0.94 (0.81–1.0) for presence of DCI when considering day 14 *HO-1* expression levels (*p* = 0.0036). *AUC* area under the curve, *CSF* cerebrospinal fluid, *CT* computed tomography, *DCI* delayed cerebral ischemia, *HO-1* hemoxygenase-1, *mRS* modified Rankin Scale, *ROC* receiver operating characteristic, *SAH* subarachnoid hemorrhage, *VSP* vasospasm

When analyzing patient subgroups, patients that exhibited sonographic vasospasm at any point in time during hospital treatment showed a nonsignificant trend toward lower *HO-1* mRNA expression levels on day 7 after SAH than those without sonographic vasospasm [Fig. 6a, *HO-1* mRNA expression level 282.1 (89.15–571.5) vs. 634.8 (224.4–1326) (fold induction vs. control), *n* = 50, *p* = 0.059]. Regarding vasospasm detected on CT angiography, patients with manifest moderate or severe vasospasm on CT angiography had significantly lower CSF *HO-1* expression levels on day 7 after SAH, compared with those who either received a CT angiography that did not show relevant vasospasm and those in which no CT angiography was performed [Fig. 6b, *HO-1* expression level 119.5 (18.06–346.9) vs. 466.3 (199.7–1004) (fold induction vs. control), *n* = 53, *p* = 0.010]. ROC curve analysis showed an AUC of 0.73 with a 95% confidence interval of 0.57 to 0.89 (*p* = 0.01, Fig. 7a). At a sensitivity of 78.57% and specificity of 64.1%, predictive power was insufficient (positive predictive value (PPV): 56.41%, negative predictive value (NPV): 44.29%, *HO-1* expression level cutoff > 301.5). There was no difference in *HO-1* mRNA expression levels on day 14 after SAH in patients with or without vasospasm (sonography: *n* = 19, *p* = 0.875, CT angiography: *n* = 21, *p* = 0.287).

In contrast, patients with DCI had significantly higher *HO-1* mRNA expression levels on day 14 post SAH, compared with patients without a DCI diagnosis [*HO-1* expression level 1777.0 (1348.0–1988.0) vs. 410.0 (90.48–755.4) (fold induction vs. control), *n* = 19, *p* = 0.009, Fig. 6c]. ROC curve AUC was 0.87 with a 95% confidence interval of 0.69 to 1.0 (*p* = 0.01, Fig. 7b). At a cutoff value of > 1254, day 14 HO-1 expression levels featured a sensitivity of 83.33%, a specificity of 92.31%, a PPV of 48.51% and an NPV of 52.05%. Further analyzing patient subgroups, patients who developed sonographic vasospasm and DCI had significantly higher *HO-1* expression levels on day 14 than those who developed vasospasm, but no DCI [*HO-1* expression level 1777.0 (1348.0–1988.0) vs. 410.0 (106.1–679.9) (fold induction vs. control), *n* = 17 *p* = 0.002, Fig. 6d]. This subgroup of patients with sonographic vasospasm showed an AUC of 0.94 with a 95% CI of 0.81 to 1.0 (*p* = 0.004, Fig. 7c). At a cutoff > 857.6 (fold induction vs. control), sensitivity of *HO-1* expression was 83.33%, specificity 100%, PPV 46.42%, and NPV 54.13%.

*HO-1* mRNA expression levels did not differ between patients that died during hospital treatment and those that could be discharged from hospital (*p* = 0.592, *n* = 53 on day 7 after SAH, data not shown).

## Discussion

The aim of this study was to examine whether *HO-1* expression levels in CSF and blood could represent prognostic markers that would indicate disease course, association with SAH complications and short-term and long-term functional outcome as early as on day 1 after SAH. In this single-center biomarker observational study, we confirmed that *HO-1* mRNA expression levels in the CSF of patients with SAH correlate with intracranial hematoma size. We demonstrated that *HO-1* expression rises over the course of the study observation period (2 weeks) after the SAH event. This study’s results do not support prognostication of functional neurological outcome based on *HO-1* expression levels. However, we found evidence that high CSF *HO-1* expression levels after one week of treatment were associated with lesser incidence of vasospasm, while late *HO-1* induction (on day 14 after SAH) was associated with higher incidence of DCI. Associations of *HO-1* expression with vasospasm and DCI need to be viewed with caution however as the small group sizes in this study did not allow for the consideration of covariates such as WFNS, age, and hematoma volume.

Our findings indicate that the semiquantitative measurement of total intracranial hematoma volume on the initial CT scans is an accurate indicator of disease severity and also reflects short-term functional development. The measurement of intracranial hematoma volume on a continuous scale rather than the traditionally employed categorical scales such as the modified Fisher scale allowed for finer discrimination when examined in relation to *HO-1* induction.

In this study, CSF *HO-1* expression on days 7 and 14 after SAH significantly correlated with total hematoma volume, corroborating previous results obtained from a smaller subgroup of the same patient collective [19]. Preclinical studies have demonstrated upregulation of *HO-1* expression as early as 7 h after ICH [35], whereas *HO-1* expression peaked at around 3 to 7 days [36, 37]. The lack of association between CSF *HO-1* expression on day 1 after SAH and hematoma volume in our patient collective may reflect the delayed peak in *HO-1* expression/HO-1 activity after hemorrhage, in line with the existing preclinical data on ICH [24, 37, 38].

*HO-1* expression levels in blood leukocytes showed no association with CSF *HO-1* expression or functional outcome parameters. In general, blood analyses have the distinct advantage of being far more readily available than CSF samples throughout the disease course and in a greater number of patients with SAH. The lack of correlation between CSF and blood leukocyte *HO-1* expression levels regarding both the extent and temporal dynamic of *HO-1* induction points to central nervous system microglia as being the principal mediators of *HO-1* induction. This supports the hypothesis that upregulation of *HO-1* expression following SAH is confined and thus specific to the central nervous system and does not involve systemic immune responses.

Preclinical models of ICH have demonstrated that the temporal course of *HO-1* induction may be crucial regarding its function; one study showed toxic effects of early *HO-1* upregulation after ICH [38], whereas late sustained upregulation exerted neuroprotective effects, while others proposed antioxidant, protective properties of early *HO-1* induction during the first days after experimental ICH and prooxidant, detrimental effects of late sustained *HO-1* upregulation [36, 38]. Our clinical data suggest that there may be detrimental effects of late sustained *HO-1* upregulation after SAH.

In the nonfavorable outcome group, day 14 CSF *HO-1* levels were higher than in the favorable outcome group. In terms of predictive ability, the multiple logistic regression model that included CSF *HO-1* day 14 expression levels as predictor variable was superior to the model without CSF *HO-1* levels and to the model with CSF *HO-1* day 7 levels. However, CSF *HO-1* day 14 expression levels did not reach significance as predictor variable in the multivariate model and WFNS could not be included in the model because of multicollinearity, limiting clinical applicability. It must be considered that this study was not powered to identify a nonassociation of *HO-1* expression levels and clinical outcome, so that these results need to be viewed with caution. It must be taken into account that the number of patients for whom CSF samples were available on day 14 was limited (*n* = 21) and that those patients were more severely affected than the overall study population (Hunt & Hess Grade 3.45 vs. 3.2 in the overall study population, mRS at discharge 4.25 vs. 3.5, 1-year mRS 3.2 vs. 2.8, total hematoma volume 33.02 vs. 22.8 cm^3^). It is likely that higher *HO-1* mRNA expression levels in patients with unfavorable outcome are an expression of disease severity and higher intracranial hematoma burden that mediates prolonged *HO-1* induction. Rising *HO-1* induction over the course of multiple weeks could be an indicator of ongoing microglial activation and thus neuroinflammation as one of the mediators of poor outcome in this patient subgroup. In a homogenous patient population consisting of patients with exclusively Fisher Grade III SAH, high HO-1 protein levels in the CSF on day 7 after SAH were associated with more unfavorable 3-month outcome [39]. Our data showed an association of higher *HO-1* expression levels on day 14, but not day 7, with more unfavorable functional outcome. Considering the homogeneity of this study population further suggests that *HO-1* induction has a component that may be independent of disease severity, although these results warrant confirmation in a larger study population.

EVD-associated complications occurred in 13.6% of the study population (ventriculitis: *n* = 5, intraparenchymal hematoma: *n* = 4). It must be taken into account that management-associated complications may influence functional short-term and long-term outcome and thus limit interpretability of outcome data.

The relationship between sonographic or angiographic vasospasm and delayed cerebral ischemia remains ambiguous, with DCI (even if showing a distinct vascular distribution pattern) not necessarily being associated with manifest vasospasm and vice versa [5]. Therefore, associations between the occurrence of DCI and vasospasm have to be made with caution.

In our study population, patients with sonographic or CT angiographic vasospasm showed lower CSF *HO-1* levels on day 7, but not on day 14 after SAH, suggesting that early *HO-1* induction may exert a protective effect on the development of vasospasm. In contrast, patients who developed DCI during the disease course had significantly higher *HO-1* expression levels on day 14 after SAH, independent of the presence of sonographic vasospasm. However, *HO-1* expression levels did not have sufficient predictive power with regards to vasospasm and DCI, limiting clinical applicability. As high *HO-1* expression on day 14 was also associated with unfavorable functional outcome as discussed above, it should be further investigated whether prolonged *HO-1* induction in the CSF could be an indicator of sustained neuroinflammation and thus increased neuronal injury, despite necessity of sufficient *HO-1* induction for hematoma clearance.

In patients with SAH and vasospasm, HO-1 protein has been found to be increased compared to the CSF of patients with SAH without vasospasm [40]. The authors propose that increased *HO-1* activity leads to increased formation of bilirubin and bilirubin oxidation products (BOXes) which increase the likelihood of vasospasm because of oxidative stress, despite availability of the same amount of substrate (hemoglobin) in the CSF. However, while CSF bilirubin concentration was demonstrated over a period of 10 days after SAH, this study did not specify the time-point of *HO-1* determination in the CSF, so that taking a possible temporal induction pattern into account, it is unclear whether our data are contradictive.

## Conclusions

Our study confirms that there is a distinct temporal induction pattern of *HO-1* expression in the CSF of patients with SAH with an extent dependent on intracranial hematoma burden that cannot be observed in peripheral blood. *HO-1* expression levels over the first week after SAH were unable to predict short- and long-term functional outcome. Patients with vasospasm showed lower CSF *HO-1* expression levels on day 7, whereas late sustained *HO-1* expression was associated with higher incidence of DCI, pointing to possible detrimental effects of sustained *HO-1* expression during the second week after the SAH event. However, the small sample size of CSF samples on day 14 after SAH necessitates further validation in a larger study population.
